# Cesarean Scar Ectopic Pregnancy: Diagnosis With Ultrasound

**DOI:** 10.5811/cpcem.2019.10.43988

**Published:** 2020-01-15

**Authors:** Taryn Hoffman, Judy Lin

**Affiliations:** Maimonides Medical Center, Department of Emergency Medicine Brooklyn, New York

## Abstract

We present a rare case of cesarean scar ectopic pregnancy as diagnosed by transvaginal ultrasonography. Cases such as this are rare, but they are becoming more commonly detected with the growing frequency of cesarean sections, improving technology, and provider proficiency with point-of-care ultrasound. Quick identification of this dangerous diagnosis can be life saving for the patient, as the outcomes of ruptured cesarean ectopic pregnancy may include significant hemorrhage, uterine rupture, and possibly maternal death.

## INTRODUCTION

The rates of cesarean deliveries have shown a steady increase over the past few decades.^1^ Given this increase and the improved technology of sonographic imaging, the incidence of detection of cesarean scar ectopic pregnancies has also shown a rise.^1^ A cesarean scar pregnancy (CSP) is a developing pregnancy implanted in the myometrium of a previous cesarean delivery scar.^2^ Although CSP is still a rare diagnosis, it is of critical importance for practitioners to be able to quickly identify and intervene on this dangerous condition. Undiagnosed CSP may progress to uterine rupture, hemorrhage, loss of future fertility, and possibly maternal death.

Overall mortality has trended downwards, but unrecognized ectopic pregnancy of any abnormal location remains a significant cause of pregnancy-related death.^3^ Ectopic pregnancies comprise approximately 2% of all pregnancies.^2^ Treatment for a diagnosed CSP may include both medical and surgical options, with the best option likely being a combination approach.^4^,^5^ Diagnosis is most rapidly made by ultrasound. We present a rare case of cesarean scar ectopic pregnancy as diagnosed by transvaginal ultrasonography.

## CASE REPORT

A 28-year-old female gravida 6 para 3023 with a last menstrual period six weeks prior presented to the emergency department (ED) for vaginal bleeding. She denied any fever, chills, abdominal pain, nausea, vomiting, chest pain, or dizziness. She had a past surgical history of three prior cesarean sections. Her urine beta human chorionic gonadotropin (ß-hCG) was positive in the ED, and her serum quantitative ß-hCG was 19,175 milli-international units per milliliter (mIU/mL) (reference < 5 mIU/mL). A transvaginal point-of-care ultrasound (POCUS) was performed, revealing a gestational sac with a yolk sac located on the anterior aspect of the lower uterine segment (Video). The endomyometrial mantle was measured at 0.35 centimeters and therefore concerning for an ectopic pregnancy (Image). Other ultrasound findings of cesarean scar ectopic pregnancy are depicted in the figure and table below.

The obstetrics and gynecology (OB/GYN) team was consulted and their repeat transvaginal ultrasound confirmed the abnormal location of the yolk sac, which was concerning for a CSP. Due to her lack of abdominal pain, the patient was discharged home and instructed to follow up at the OB/GYN clinic the next day. On next day follow-up, she received an ultrasound showing an unruptured CSP and received a dose of intramuscular methotrexate at one milligram per kilogram (mg/kg). She was instructed to return the day after to receive a multi-dose regimen of methotrexate alternating with intramuscular leucovorin at 0.1mg/kg, but did not return for repeat dosing. She was contacted one month later and reported that she no longer had any abdominal pain or vaginal bleeding.

## DISCUSSION

Cesarean scar ectopic pregnancies are rare, comprising less than 1% of all pregnancies.^2^ In recent years, the incidence has increased due to the growing frequency of cesarean sections. The Centers for Disease Control and Prevention (CDC) reported a cesarean section rate of 20.7% in 1996, which grew to 32% in 2017 in the United States. This increase in rate of CSP detection may also be due to improvements in image quality of transvaginal ultrasound as well as the increasing use of transvaginal POCUS. This uncommon condition was first described by Larsen and Solomon in 1978, with only 19 additional cases documented until 2001.^7^,^8^ It now accounts for nearly 5% of all ectopic pregnancies in women with prior cesarean deliveries.^9^

Ultrasound is the initial imaging test of choice for diagnosis of CSP with a sensitivity of 86.4%.^8^ When evaluating a first trimester pregnancy by transvaginal ultrasound, there are multiple criteria used to diagnose CSP (Table).

A CSP is diagnosed when the uterine cavity and cervical canal are empty and the gestational sac is in the anterior portion of the uterine isthmus.^8^ The thickness of the myometrium at the site of implantation is thin; this can be measured at the site between the gestational sac and the bladder, and is abnormal when less than eight millimeters.^5^,^11^ Approximately two-thirds of cases of CSP have a myometrial thickness less than five millimeters.^6^ This abnormal implantation occurs when the blastocyst implants into the scar tissue from a prior cesarean incision; it invades into the remaining tract from the prior uterine wall disruption.^12^ Women who have had multiple cesarean deliveries carry a higher risk of abnormal implantation into the fibrotic scar tissue.^6^

There are two types of CSP, differentiated by the depth of invasion. The first type is implanted deeply into the scar defect, up to the serosal lining and possibly into the bladder or abdominal cavity. This type is very dangerous; it has a high risk of uterine rupture and hemorrhage.^13^ The second type implants in the scar but grows away from the serosal lining and toward the uterine cavity.^2^

When evaluating a pregnant patient with vaginal bleeding or abdominal pain, it is important to consider ectopic pregnancy, abnormally invasive placenta and spontaneous abortion.^14^ Taking a thorough history including outcomes of all prior pregnancies is crucial. When considering CSP, cervical ectopic pregnancy and abortion in progress should also be included in the differential. A cervical ectopic pregnancy will have the gestational sac implanted in the cervix, with the sac located in the endocervical canal rather than embedded in the anterior lower uterine segment. This may look similar to a CSP, but the anterior myometrium will be of normal thickness. When examining an abortion in progress, the cervical os may be open, the anterior myometrium will also be of normal thickness, and the fetus may be seen within the cervical canal without fetal cardiac activity. The cervical os is closed in a CSP, so the pelvic exam is useful to help further differentiate these diagnoses.^9^, ^15^

**CPC-EM Capsule**

What do we already know about this clinical entity?

A developing pregnancy can implant into the preexisting scar from a prior cesarean section delivery. Delayed diagnosis could lead to hemorrhage and maternal death.

What makes this presentation of disease reportable?

We present a rare cause of ectopic pregnancy with abnormal sono-anatomy identified through point-of-care ultrasound (POCUS), leading to expedited consult and diagnosis.

What is the major learning point?

Cesarean scar pregnancy (CSP) should be in the differential diagnosis for any pregnant patient with abdominal pain or bleeding. POCUS findings can raise suspicion for CSP.

How might this improve emergency medicine practice?

Educating providers on POCUS findings consistent with CSP may help to avoid misdiagnosis and expedite obstetrics consultation and appropriate management.

Currently there are several acceptable methods of treatment for CSP. A retrospective chart review conducted by Riaz, et al. of 20 women with CSPs found that these patients were treated with a combination of intramuscular methotrexate, local embryocidal methotrexate injection, or surgery.^15^ Five of the 15 patients who received methotrexate had successful abortions, although three of those patients required additional doses. Although single-dose therapy has been found to be effective in some cases, it is likely that patients will require additional doses or more invasive treatments for successful termination of the ectopic pregnancy. There is no widely accepted consensus on the matter; practice patterns vary based on patient, provider, and facility in which the treatment takes place. The goals of therapy are the same, however, which are to prevent dangerous blood loss or uterine rupture while preserving the woman’s fertility for future conceptions.^15^ When methotrexate fails or is not an option, the next-line therapy is laparoscopic resection.^16^

## CONCLUSION

Ultrasonography has become a vital skill in the scope of practice for emergency physicians. It allows for rapid identification of potentially life-threatening conditions. This case report reveals an interesting and rare case of CSP as diagnosed by transvaginal ultrasound. Although diagnosis of CSP on ultrasound may be outside the scope of practice for emergency physicians, it is still important to be aware of CSPs and they should be within the differential of any pregnant patient presenting with abdominal pain or vaginal bleeding with a history of prior cesarean section. Knowledge of the criteria for CSP can help emergency physicians to be familiar with findings of an abnormal pregnancy on ultrasound, which should prompt additional imaging and urgent consultation from an obstetrician.

## Supplementary Information

Video. Ultrasound clip of cesarean scar ectopic pregnancy with narration. Indicator shows relevant anatomy: empty uterus with a clearly visualized endometrium; empty cervical canal; and gestational sac implanted in the anterior lower uterine segment with a thin myometrium measured at 0.35 centimeters.^6^

## Figures and Tables

**Image:**
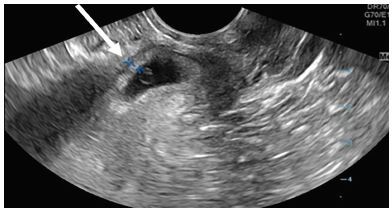
Transvaginal ultrasound view of uterus in sagittal plane showing cesarean scar ectopic pregnancy. Myometrium thickness of anterior uterine segment (arrow) measures 0.35 centimeters, which meets diagnostic criteria for ectopic pregnancy.^6^

**Figure:**
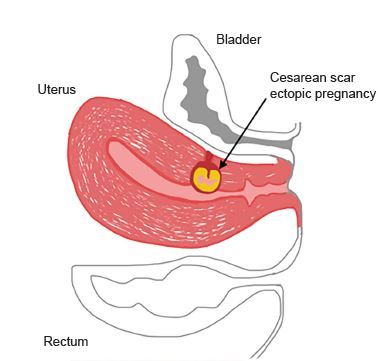
Anatomic depiction of uterus with cesarean scar ectopic pregnancy. Features of cesarean scar ectopic pregnancy seen here include an empty uterus, gestational sac implanted in lower anterior segment of uterus with thin myometrium and empty cervical canal.^10^

**Table:** Ultrasound criteria for diagnosis of cesarean scar ectopic pregnancy.^6^

Empty uterus with clearly visualized edometrium Empty cervical canal Gestational sac implanted in the lower anterior uterine segment at the presumed site of cesarean section incision scar Thin or absent myometrium between the gestational sac and the bladder. (Majority of cases have a myometrium thickness < 5 millimeters)
